# Diseases of the Arteries, Capillaries, and Veins

**Published:** 1892-01-09

**Authors:** 


### DISEASES OF THE ARTERIES, CAPILLARIES AND VEINS.—I.

In this paper the causation of these affections will be principally considered, and a classification will be given in accordance with their origin. The subject may be arranged under three heads.

1.—THE ARTERIES.

The remarkable immunity of these vessels from destructive diseases has often been noticed. In chronic abscesses an arterial branch frequently survives whilst all its surroundings have broken down and become purulent, and it is well known that the disorganising processes which go on in phthisical lungs spare the arteries in a marked manner. This is no doubt due partly to their tough, elastic nature, and partly to the constant renewal of their contained blood. Their vital properties are well shown by the power they possess of sending out new vessels, as in the healing of wounds and granulating surfaces. Of all their coats, the fibrous tissue is the most prone to be affected, especially the so-called subendothelial tissue, and next to this the external coat. In the former commence the changes included under the name endarteritis; in the latter periarteritis takes origin.

Traumatic causes need not be considered. A severe enough injury occasions inflammation or destruction, as in other parts of the body. Extension of disease from neighbouring tissues, as in cellulitis, &c., calls for no special remarks. It is sufficient for our purpose to notice (1) that acute periarteritis is probably always traumatic, or due to extension from neighbouring inflammations, and (2) that the middle coat is the last to be involved in such circumstances, chiefly because of its poorness in connective tissue elements, and because of its protected position between the internal and external elastic laminæ, the latter existing to some extent in most arteries.

The other forms of periarteritis are the *tubercular* and *syphilitic*. These are both chronic, and only exist in connection with disease of the other coats.

It is probable that no inflammatory changes begin in the middle tunic, but the muscular tissue is hypertrophied in many different conditions, especially in Bright's disease and in syphilis. In the latter it is distinctly secondary to endarteritis. In the former it is preceded by a little thickening of the subendothelial tissue, but it is by far the most pronounced feature. It is due, undoubtedly, to the contraction of the circular fibres, and this, in its turn, seems to be brought about by the irritation of the urea, uric acid and their antecedents circulating in the blood, elimination of the nitrogenous metabolites being, of course, defective. Constant contraction leads to permanent thickening, as in other muscular organs. There is frequently a great deal of fibrous tissue mixed with the new unstriped fibres, as in similar conditions in other parts.

This affection must begin in the very smallest arterioles, or in the capillaries themselves. Dr. Howship Dickinson points out (in the Hunterian Oration, 1891) that the rupture of the minute vessels of the retina in Bright's disease indicates obstruction beyond these, and that œdema of the tissues from constriction *a fronte* must be in the capillaries, inasmuch as exudation only takes place through these and through the microscopic arteries.

The middle coat is affected secondarily in most of the arterial diseases. Fatty degeneration of the muscular fibres occurs in old age, and as an accompaniment of the same change in the heart. This leads sometimes to calcareous patches, which must be considered as distinct from ordinary atheroma.

The internal coat plays the most important part in arterial disease, and most, if not all, of its affections may be traced to blood conditions. The endothelial lining consists of elongated cells with sinuous outlines, nucleated and pliable. Under this is the subendothelial tissue, which has a structure on a minute scale somewhat resembling that of the cornea, that is to say, there are laminæ of fibrous tissue, longitudinal and transverse, with small, branched, connective tissue cells in the meshes. It is in this that the changes of endarteritis are most marked. Acute inflammation of this tissue sometimes follows the impaction of emboli, which have become infected and contain germs. This naturally occurs in the smaller arteries, but if the lining of the vessel is gone (as in early stages of atheroma) the materies morbi may cause localised patches of disease in the larger trunks.

*Atheroma*, which is almost always a chronic change, begins as a thickening in the subendothelial tissue, accompanied by a shedding of the epithelioid cells. Inflammatory products accumulate, and a softened patch is the result. The disease usually spreads to the other coats of the vessel. What originates this disease? It is said to be most common in France and Great Britain, and to be rare in countries where the consumption of alcohol is small. It is possible, therefore, that the irritation of this substance circulating in the blood is the cause of the changes. It is probable, however, that the general deterioration of tissue and diminished oxidation that result from the abuse of alcohol are more important factors than the actual irritation.

*Endarteritis obliterans* is subacute rather than chronic, and the form which occurs in the healing of wounds, and as a result of injury, is acute.

In Bright's disease, and the condition described as arterio-capillary fibrosis, the changes begin as endarteritis, but seldom lead to "obliteration," and should not, strictly speaking, come under this head. In tuberculosis of various organs the disease is often pronounced, complete blocking of the lumen occurring, as may be seen in tubercular testis and in tubercular meningitis. In fact, a well-known German pathologist asserts that the "giant cells" frequently take origin in small arteries and capillaries, the endothelial coat remaining as a characteristic part of the typical giant-cell capsule.

But perhaps the most marked examples of the affection are in syphilis. M. Huchard, in a clinical lecture delivered at the Hôpital Bichat (July, 1891), describes three stages in syphilitic arterial disease, viz., (1) Induration, (2) vascular stenosis with ischæmia of the part supplied by the vessel, and (3) complete obliteration of the lumen, with gangrene or sclerosis of the tissues. The condition is often quite localised, but may be general. Both in this and in tubercular disease the adventitia is thickened. It can hardly be doubted that some distinct poison circulating in the blood is the cause of these changes. The part played by the narrowing of the vessels is very important; the frequency of necrosis, caseous degeneration, and fibroid thickening of various parts in syphilis may all be accounted for by this one factor.

Amyloid disease will be considered under "the capillaries." We thus see that disease of the arteries usually begins in the fibrous part of the intima, or in the adventititia. The only primary affection of the middle coat is probably fatty degeneration, and perhaps hypertrophy. *Periarteritis* is either traumatic or secondary to disease in other coats of the vessels, as in syphilis and tuberculosis.

The internal coat is primarily involved in atheroma, syphilitic disease, tuberculosis, and Bright's disease; also in acute endarteritis from septic infection.

